# Bridging production and protection: Legislative and technical feasibility of continuous cover forestry around freshwater in Sweden

**DOI:** 10.1007/s13280-025-02340-4

**Published:** 2026-01-23

**Authors:** Irina Mancheva, Alejandro Gándara, Luis Andrés Guillén, Lenka Kuglerová, Anneli M. Ågren, Francisco X. Aguilar, Eliza Maher Hasselquist

**Affiliations:** 1 Department of Political Science, Umeå University, 901 87 Umeå, Sweden; 2 Department of Forest Ecology and Management, Swedish University of Agricultural Studies (SLU), Skogsmarksgränd 17, 90183 Umeå, Sweden; 3 Southern Swedish Forest Research Centre, Swedish University of Agricultural Studies (SLU), Sundsvägen 3, 234 56 Alnarp, Sweden; 4 Department of Forest Economics, Swedish University of Agricultural Studies (SLU), Skogsmarksgränd 17, 90183 Umeå, Sweden

**Keywords:** Continuous cover forestry (CCF), EU Nature Restoration Regulation, Forest riparian buffers, Land-use policy goals, Legislative feasibility, Technical feasibility

## Abstract

Forests are essential to the global green transition, but intensified management may undermine environmental goals. Continuous cover forestry (CCF) offers an alternative, balancing timber production and ecosystem conservation. We investigated the legislative and technical feasibility of CCF in riparian buffers along freshwaters, where it could potentially restore simplified stands and better protect water. We analyzed EU, national, and global policies identifying barriers and enablers and used GIS data from 11 Swedish sites estimating the share of productive forest converted to CCF under varying buffer widths. Results show CCF in riparian buffers is feasible, with few legislative barriers and several soft enablers. The EU Nature Restoration Regulation is a key enabler, as CCF-managed buffers can contribute to restoration targets, providing opportunities for different property sizes to meet multiple management objectives. Future feasibility depends on EU member states’ implementation of the regulation and alignment of ecological goals with political and economic conditions.

**Supplementary Information:**

The online version contains supplementary material available at 10.1007/s13280-025-02340-4.

## Introduction

Forests and forestry will play a pivotal role in climate change mitigation and the global green transition (Grassi et al. 2017; Gregor et al. 2024). However, there are concerns that forest management toward advancing these goals may lead to an increase in harvest intensity in Europe and a disbalance between production and protection goals (EC 2019; Blattert et al. 2022; Chapman et al. 2025). A growing body of research has underscored the potential of less intensive forestry practices, e.g., primarily selective logging often called “continuous cover forestry” (CCF), as an alternative to intensive rotation forest management in attaining multiple objectives, including timber production, biodiversity protection, climate change mitigation, and water and wetland conservation goals (Knoke 2012; Eyvindson et al. 2021).

Forest management practices, such as clear-cutting (also “rotation forestry”), can have a direct and acute impact on freshwater quality, quantity, and biodiversity (Eklöf et al. 2014; Oldén et al. 2019). These impacts can be ameliorated by implementing riparian buffers, a protection measure of leaving forested strips unharvested around water (Kuglerová et al. 2024). Riparian buffers are at the ecological and policy interface between aquatic and terrestrial systems (Hasselquist et al. 2020; Urbanič et al. 2022) and can also serve as green corridors connecting habitats across the landscape (Salviano et al. 2021), supporting multiple land-based environmental goals. Sweden, one of Europe’s most forested countries and a leading timber-producer (FAO 2020), lacks regulations setting concrete and measurable requirements for the adoption of riparian buffers, which likely explains their limited implementation, especially around the smallest waterways (Hasselquist et al. 2020; Kuglerová et al. 2020; Ring et al. 2022; Kuglerová et al. 2024). However, increased protection could place an uneven degree of financial strain on different landowners (Bostedt et al. 2021; Bakx et al. 2024), making riparian buffer implementation complex. Apart from allowing production-oriented forestry while simultaneously preserving key ecosystem services, active management of forest riparian buffers through CCF could also ameliorate the negative effects of historical management decisions that have led to single-storied, single-species riparian forests (Hasselquist et al. 2021).

Despite that CCF implementation became possible in Sweden after the removal of restrictions on its application in production forests with the Forestry Act of 1993 (Sténs et al. 2019), a lack of sectoral acceptance together with a lack of momentum in implementation is still hampering CCF’s diffusion in practice as opposed to conventional, more intensive management methods (Hertog et al. 2022; Dawson et al. 2025). Management practices can be changed through the enactment of different policy instruments or mixes of such (Rogge and Reichardt 2016; Tesfaye et al. 2024), where regulatory instruments play a vital role (de Boon et al. 2021). Yet, the political and technical feasibility of policy is critical for successful implementation, behavioral change, and ultimately, to attain political goals (Kingdon 1984; Shephard et al. 2021). Given that the recent legislative changes at the European Union (EU) level, e.g. the enactment of the EU Nature Restoration Regulation (2024/1991), have altered the conditions for the implementation of CCF across European productive forests, our study explores the legislative and technical feasibility of CCF adoption in riparian buffers, as a tool that can help reconcile different policy goals.

Legislative feasibility refers to the institutional frameworks (Majone 1975) in the form of already-enacted global (FSC), supranational (EU) and national (Swedish) policies, and their role in obstructing or incentivizing CCF adoption. Since we analyze the feasibility of a land management practice and not of a proposed policy, we refrain from the more traditional definition of political feasibility (e.g., policy acceptance by actors at each stage of the policy-making process; Webber 1986). Instead, we explore the *legislative feasibility* of CCF in relation to existing forest and freshwater policy requirements. *Technical feasibility* is explored by probing the potential of CCF to attain the multiple goals of forest and freshwater policies in different riparian width scenarios in  productive forests, or if CCF “will work” (Shephard et al. 2021, p. 525). We do this by estimating the percentage of productive forest that would be included in different buffer scenarios and managed through CCF in eleven case study areas across Sweden. We focus our discussion on CCF implementation in a Swedish context to stress the complexity of navigating both legislative and technical feasibility of alternative forest management practices in countries with a strong forest sector, while also providing valuable lessons to policymakers in a broader international context.

### Forest management in Sweden and continuous cover forestry

Forest management in Fennoscandia has historically favored conifers (e.g., Norway spruce *Picea abies* and Scots pine *Pinus sylvestris*) reducing broadleaved species and altering natural disturbance regimes such as fire and canopy gap dynamics, thus affecting freshwater, among other ecosystem services (Esseen et al. 1997; Linder et al. 1997; Hellberg et al. 2009; Kritzberg et al. 2020). Dense, single-story, conifer-dominated riparian forests can excessively shade streams and riparian areas, limiting understory plant diversity and stream productivity (Burrows et al. 2021; Myrstener et al. 2025). Introducing more broadleaved species to riparian zones can improve light and microclimate conditions, enhance nutrient retention, and support diverse aquatic food webs (Lidman et al. 2017). Mixed-species riparian forests also enhance biodiversity, including rare species and macroinvertebrates (Bell et al. 2015). Management practices that mimic natural disturbances, such as selective logging or gap cutting, could further promote ecological functioning and biodiversity while protecting freshwater quality and quantity (McKie and Malmqvist 2009; Sibley et al. 2012).

The Swedish Forest Agency defines CCF (termed as “*hyggesfritt”* or *non-clear-cut forestry*) as a management practice in which the land is always forested. CCF relies on different methods such as gap cutting, selective cutting, shelterwood or checkboard cutting to maintain a continuous forest canopy (Appelqvist et al. 2021). Stipulations include maintaining a long-term perspective, keeping 10-m tall trees within the forest stand, ensuring the volume of the stand does not fall below a specified level to maintain production goals, and limiting gap cuts to no larger than 0.25 ha. This is in direct contrast with traditional rotational forestry, or clearcut methods implemented in Sweden where large areas (regional averages 2.5–5.6 ha) are harvested at once, leaving the area without a forest canopy until the canopy closes again (about 30–60 years across Sweden; Kuglerová et al. 2020).

## Conceptual framework: Change in land management and the feasibility of alternative forestry practices

### Legislative feasibility: Regulatory instruments as barriers to or enablers of change

Change from conventional to alternative land management practices hinges on local and global social, technological, economic, and cultural factors (Ferrer Velasco et al. 2023; Chapman et al. 2025). Policy studies recognize that major policy shifts are typically rare, and more limited changes materialize when interventions addressing well-defined issues (in our case CCF in riparian forest management) target specific action(s) and actors through policy instruments (Cairney and St Denny 2020). Policy instruments can be categorized into regulatory, economic, and informative (Bemelmans-Videc et al. 2011). Some actors may perceive regulatory policy instruments alone as ineffective (Ferrer Velasco et al. 2023), but regulation is needed to trigger change in management practices (Rogge and Reichardt 2016; de Boon et al. 2021) and forms the basis for the use of other policy instruments, e.g., economic (Lambin et al. 2014). Regulatory policy instruments can be further categorized into whether they incentivize or disincentivize a certain behavior (Bemelmans-Videc et al. 2011; Ferrer Velasco et al. 2023), and whether they are binding (hard) or non-binding (soft) (Treib et al. 2007). Regulatory policy instruments act as barriers to or enablers of change since they can impede or foster both the adoption of other policy instruments, as well as specific management practices. The legislative feasibility of forest management practices depends on policies from multiple sectors and institutional levels because of forests’ expected role in attaining production *and* conservation policy goals, (e.g., timber production, biodiversity conservation, climate mitigation, and bioenergy) and because goals are set at various institutional levels (i.e., international, national, local) (Mårald et al. 2017; Sotirov and Storch 2018; Chapman et al. 2025).

The Swedish case is a case in point, where forests and freshwater are governed independently (Mancheva 2020) and policies from the forest and water regulative domains can simultaneously act as barriers or enablers for the implementation of CCF in their interface: *riparian buffers*. For example, non-binding regulations in the form of recommendations for implementing CCF in riparian buffers prescribed by forest policy at the national level could be countered by binding regulation prescribed by water policy at a supranational level setting protection goals and restricting any management measures in riparian buffers. However, even if certain management is legislatively feasible (meaning that no hard barriers to implementation are identified), policy instruments may not be enough to spur change if hampered by practical/technical limitations (Geels and Gregory 2024). We thus explore not only the legislative feasibility of CCF, but also its technical feasibility.

### Technical feasibility: The ability of CCF to attain policy goals

Production, restoration, and protection often compete for the same land, potentially leading to an overestimation of the feasibility of restoration and protection measures (Chapman et al. 2025). By focusing on riparian buffers in productive forests, we can explore if CCF in different riparian buffer scenarios can attain restoration and protection goals under different policies (regulating forest and/or freshwater management), while allowing continued management for timber production within the same areas. In doing so, our study identifies not only policy barriers and enablers but also explores whether the extant policy framework supports technologically feasible practices (Roberts et al. 2012; Shephard et al. 2021; Geels and Gregory 2024) across forests with primarily production goals. We also evaluate how management changes may disproportionately burden small landowners, potentially reducing uptake and undermining policy goals (Bakx et al. 2024). Furthermore, since definitions play a significant role in policy implementation (Head 2019), we explore riparian buffer scenarios for freshwaters covered by various definitions: natural streams, lakes, and ditches separately.

## Materials and methods

**Fig. 1:**
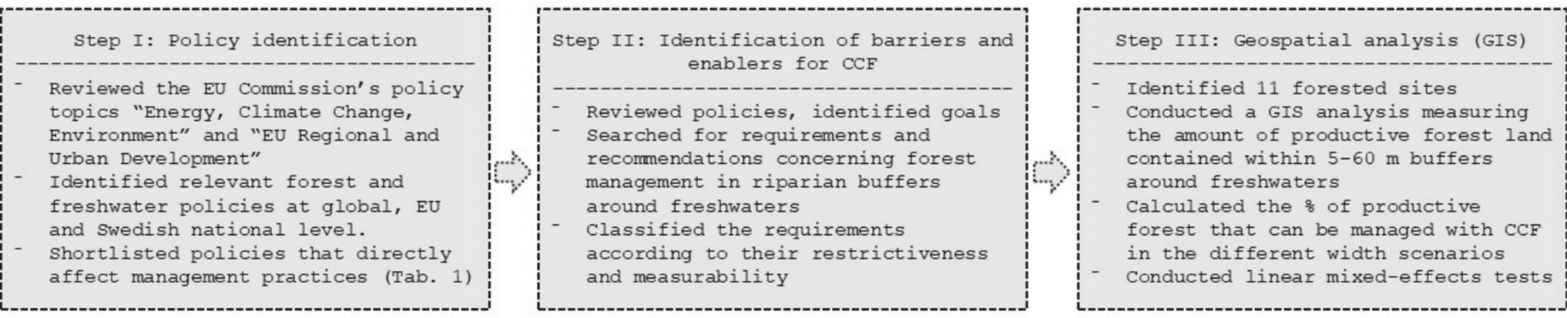
Our three-step research approach to analyze CCF’s legislative and technical feasibility

We applied a three-step research approach (Fig. 1) using qualitative and quantitative methods. The first two steps were a qualitative policy analysis exploring legislative feasibility, while the final step consisted of a geospatial (GIS) analysis to determine technical feasibility. We chose Sweden as the geographical and administrative unit for analysis, because of (1) the significance of wood production for the national economy (Sweden is the second largest wood producing country in Europe, only after Russia; FAO 2020); (2) the length of natural and modified waterways (> 1 million kilometers, Laudon et al. 2022) and lake shorelines (427 985 km shorelines SCB 2024) within Swedish forests (collectively referred to as “freshwaters” here); (3) the relatively minimal requirements set by forest regulation that leave most management decisions to the landowner’s discretion (Appelstrand 2007; Johansson and Keskitalo 2014), and few hard legal requirements specifically for management and protection of freshwaters in forests (Keskitalo and Petterson 2012; Hasselquist et al. 2020); and (4) the supranational (EU) regulation affecting the Swedish forest and water sectors.

In step one, we identified policies at the EU and Swedish national and subnational levels relevant to forest and water (Table 1). Specifically, we examined the EU Commissions’ policy topics “Energy, Climate Change, Environment” and “EU Regional and Urban Development”. We identified all policies (both hard and soft) that directly or indirectly concern forest or freshwater management (Fig. 1) and shortlisted the policies that affect management practices directly with prescriptions (Table 1). As a second step, we identified the main policy goals and those relating to riparian buffers, as well as barriers and enablers for the implementation of CCF in forest riparian buffers around freshwater: natural streams, lakes, and ditches. We did this by searching for requirements and recommendations for management or protection of forests around water. We then classified the requirements based on whether they: (a) were binding (hard) or not (soft) and b) set measurable targets. In this approach, binding requirements for set-asides in forest riparian buffers that forbid any kind of management practices were considered barriers to the implementation of CCF. However, the lack of barriers and the absence of any (binding or not) requirements for the application of CCF in forested riparian buffers would not necessarily be seen as a clear enabler.

**Table 1:** Summary of analyzed policies, their goals and whether they were identified as barriers to or enablers for CCF implementation in forest riparian buffers in Sweden. A reference list of the policies with links is available in S4 of the Supplementary Materials

Institutional level of the policy	Regulation/Policy	Main policy goals	Riparian buffers mentioned	Prescriptions for riparian buffers in connection to forestry	Binding prescriptions for riparian buffers	Barriers or enablers for CCF in riparian buffers
National: Sweden (SE)	SE:1 Environmental Code (SFS: 1998:808)	Promote sustainable development by protecting and preserving human health, natural and cultural environments, biodiversity; securing long-term good ecological, social, cultural and economic management of land, water and the physical environment; and encouraging re-use and recycling	Shore protection: 100 m but only for streams > 2 m. Agriculture and forestry excluded	No, although the protection of key habitats (“biotopskydd”) could limit management in forest riparian buffers	No. Potential hinder to CCF in cases when there are key habitats in riparian buffers	May act as barrier
	SE:2 Forestry Act 1979 and 1993 amendment (SFS; 1979:429; SFS: 1993:1096)	Ensure that the forest as a national asset is managed so that it produces sustained yields while maintaining biodiversity. Production and environment put at par. Other public interests must also be considered in management	No	No, although ecosystems (including aquatic) should be considered and weigh as much as production goals	No	No
	SE:3 The Swedish Forest Agency Regulation and general guidelines concerning the Forestry Act (SKSFS 2011:7) and forest water protection (SKSFS 2013:2)	Regulations and recommendations clarifying and specifying the requirements from the Forestry Act	Yes	Yes	No	No
	SE:4 Species Conservation Regulation (2007:845)	Protecting and conserving wild species of animals and plants, implementing the EU Birds Directive, EU Habitats Directive, EU Regulation on the protection of species of wild fauna and flora by regulating trade therein and the CITES Convention	No	No specific prescriptions. Management practices could be limited in riparian buffers if protected species are to be affected	No, unless protected species are found in and affected by management of riparian buffers	May act as hard barrier
	SE:5 Government decree on the management of the aquatic environment quality (SFS 2004:660)	The management of water quality and the implementation of the EU Water Framework Directive	No	No	No. Binding legislation but requires the establishment of specific environmental norms and requirements for their achievement at local–regional level	No
	SE:6 Programs of measures for management of water districts (PoM 2022–2027)	Implementing specific actions and measures needed to attain the goals of the EU Water Framework Directive and its Swedish implementing legislation, namely improve water quality, target pollution sources, protect and enhance aquatic habitats and monitor progress	Yes	Yes, but not defined. Instead, defined in Guidelines which refer back to Skogvårdslagstiftningen – all of which are recommendatory	No, since there are no definitions of buffer requirements	No
	SE:7 Guidance for heavily modified water bodies (HVMFS 2023:12)	Provides guidance on heavily modified water as support for the implementation of Water Management Ordinance	No	No	No	No
	SE 8 Guidelines for measure-planning and norm setting for the forest sector’s diffuse pollution	Aimed primarily at the County Administrative Boards for setting environmental quality standards for water in connection to forestry	Yes	Yes, recommends “adapted forestry measures” for especially vulnerable water bodies, including wetland restoration	No	May act as soft enabler
	SE: 9 Possibilities for CCF in forestry and definition of closer to nature forestry in Sweden (2023:16)	The Swedish Forestry Agency and the Swedish Environmental Protection Agency’s joint proposal for developing the conditions for CCF and for a definition of close-to-nature forestry	Yes	At least 15 m on each side of the waterway. Max 15% of the waterway should lack a buffer	No	May act as soft enabler
	SE:10 Agency regulation for classification and environmental quality objectives for surface water (HVMFS 2019, 25)	Regulations and recommendations for the classification of water bodies and the establishment environmental quality standards by Water Authorities. Implementing the EU Water Framework Directive and the Water Management Ordinance (2004:660)	Yes, defined as areas around waterways	Yes, if water status is to be considered as “good” or “high” and for water status not to deteriorate	No. Binding but only give instructions on how to set environmental goals, no requirements for forestry per se	May act as soft enabler
	SE:11 Strategic Objectives for Environmental Consideration in Forestry (SKS 2016:12)	Setting common goals for good environmental consideration in forestry and providing guidance. Contributing to forest policy goals as well as other societal goals	Yes	Yes, 5–15 m of no-machine zone, depending on management	No	May act as soft enabler
	SE:12 Blue-Yellow-Green objective classification report (HVM 2019:18)	Providing guidance and concrete measures at regional and local level to achieve the goals of favorable conservation status and good ecological status within environmental and water management	Yes	Yes, no specified width. Management for more deciduous trees	No	May act as soft enabler
Supranational: European Union (EU)	EU:1 Water Framework Directive (2000/60/EC)	Achieving good status for all water bodies and preventing deterioration, promoting sustainable water use, reducing pollution, protecting aquatic ecosystems, encouraging public participation, and coordinating water management: across borders	Yes	No, but it is framework legislation that requires additional implementation	Binding, but gives only the frame and goals, requires further implementation by member states	No
	EU:2 Forest Strategy 2030 (COM/2021/572)	Ensuring the sustainable management and protection of forests across the European Union by, protecting and restoring EU forests, ensuring that forest management practices balance ecological, economic, and social functions; improving forest quality and quantity, supporting the forest-based bioeconomy; enhancing forest monitoring and data collection; fostering public participation and stakeholder engagement; coordinating with international efforts	No	No. Does underscore CCF as a management practice that can be used as an alternative to clear-cutting and for restoration	No	No
	EU:3 Guidelines on Closer-to-Nature Forest Management	Supports the EU Green Deal and EU Biodiversity Strategy 2020. Promotes sustainable and biodiversity-friendly forest practices through: enhancing biodiversity; improving ecosystem services; supporting climate adaptation; promoting multifunctional forests; engaging stakeholders; monitoring and evaluating the outcomes of forest management practices to ensure they meet biodiversity and sustainability goals	Yes	30 m for small streams	No	May act as soft enabler
	EU:4 Guidelines on Biodiversity-Friendly Afforestation, Reforestation and Tree Planting	Provides practical recommendations to support biodiversity-friendly practices in the implementation of the EU Green Deal by enhancing biodiversity; improving ecosystem services; supporting climate adaptation; promoting sustainable land use; engaging stakeholders; monitoring and evaluating outcomes of tree planting projects	Yes	No	No	No
	EU:5 Regulation on Nature Restoration (Regulation 2024/1991)	Restoring and enhancing ecosystems across the EU by restoring degraded ecosystems; enhancing biodiversity; supporting climate mitigation and adaptation; preventing and reducing natural disasters; promoting sustainable land and sea use; engaging stakeholders and the public. Has set targets to restore at least 20% of the EU’s land and sea areas by 2030, with the goal of restoring all ecosystems in need of restoration by 2050	Yes	One of the suggested restoration measures is the Establishment of riparian buffers, and CCF management	Yes, however, no binding targets for riparian buffers specifically	May act as hard enabler
	EU:6 The Habitats Directive (Directive 92/43/EEC)	Ensure the conservation of natural habitats and wild fauna and flora across the EU by protecting biodiversity, maintaining and restoring habitats; creating the Natura 2000 network; preventing habitat deterioration, both inside and outside Natura 2000 sites; promote sustainable development by balancing biodiversity conservation with economic, social, cultural, and regional requirements	No	No specific prescriptions. Protected habitats if in riparian buffers could limit management	No. Could be barrier if management practices are to be carried out in protected habitats	May act as hard barrier
	EU:7 The Birds Directive (Directive 2009/147/EC)	Halt the decline of bird populations and ensure their long-term survival and recovery through: protecting all wild bird species naturally occurring in the EU and their habitats; preventing habitat deterioration; establishing Special Protection Areas (SPAs), forming part of the Natura 2000 network; regulating hunting and exploitation; promoting research and monitoring: Encourage scientific research and monitoring to inform and improve bird conservation efforts	No	No specific prescriptions. Management practices could be limited in riparian buffers if protected species are to be affected	No. Could be barrier if management practices are to be carried out in areas home to protected species	May act as hard barrier
Global (GL)	GL: The FSC National Forest Stewardship Standard (FSC-STD-SWE-03–2019 SW)	Promote responsible and sustainable forest management by: ensuring that forest management practices are environmentally appropriate, socially beneficial, and economically viable; protecting and enhancing biodiversity within forest ecosystems; recognizing and upholding the rights of indigenous peoples, in forest management practices; minimizing negative environmental impacts of forestry operations, including soil and water protection; ensuring fair labor practices and community engagement; ensuring compliance with all applicable laws and international treaties	Yes	Yes, same as in Strategic Objectives	Binding certification requirements but only for certified products/owners	May act as soft enabler

**Fig. 2:**
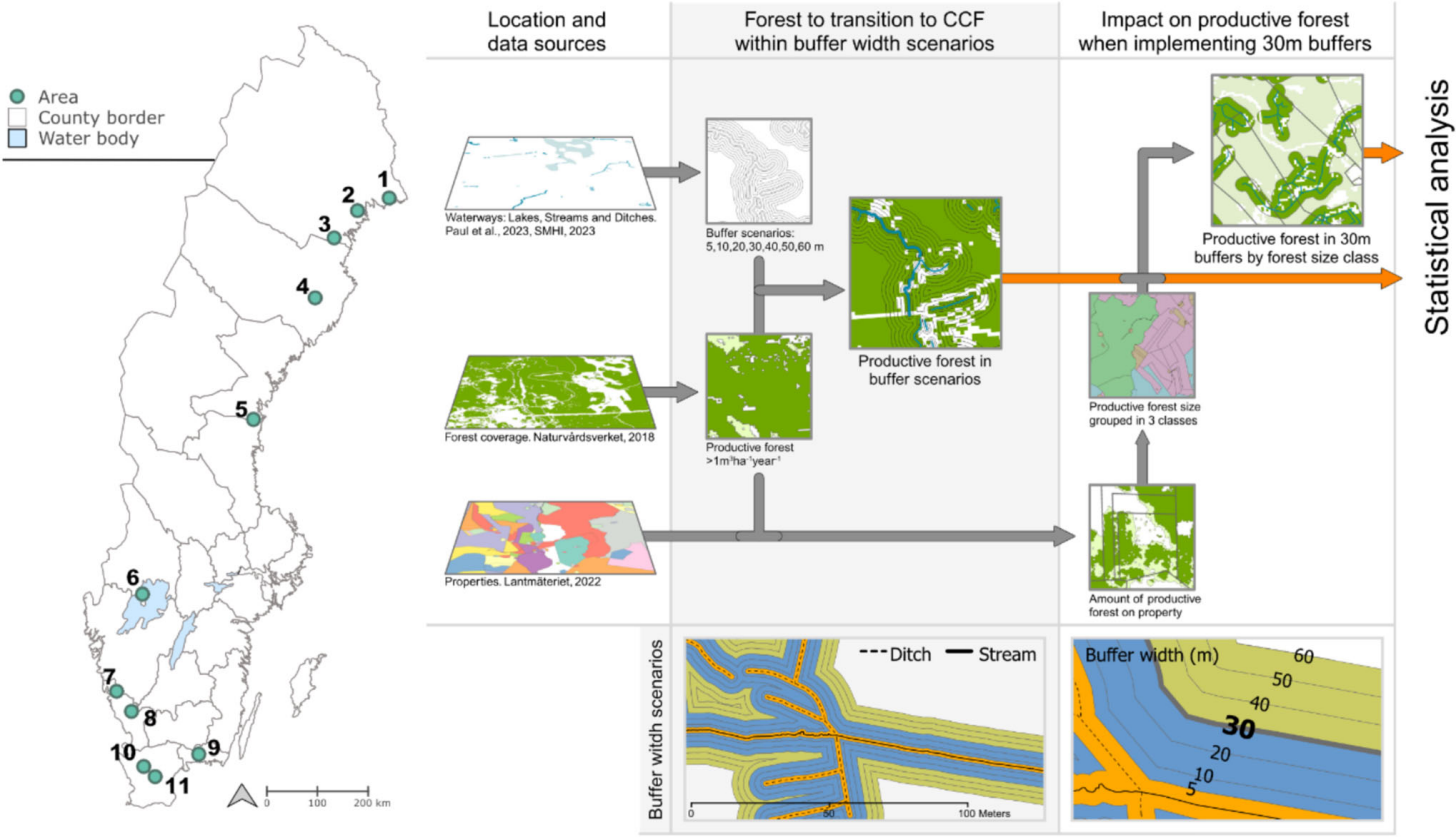
Graphical overview of data sources and GIS analysis. From left to right: Map of the 11 study areas, chosen to be representative of the Swedish forest landscape; location and data sources, and a workflow of the GIS analysis (gray arrows). Orange arrows show which data are displayed in figures and used in statistical analysis

In the third step, based on the findings from the policy analysis, we conducted a GIS analysis to determine the potential of CCF to attain the identified measurable hard and soft policy goals as a forest management measure in riparian buffers. Empirically, we included 11 sites representative of  productive forests in Sweden (Fig. 2), to infer the consequences of implementing CCF in different buffer widths (5, 10, 20, 30, 40, 50, and 60 m) on each side of all freshwaters at a national level (Fig. 2). Within the concept of “freshwaters”, we separated natural streams and ditches (Paul et al. 2023), in addition to lakes (Fig. 2). We used the forest data from the NMD-Productivity layer of the National Land Cover Database 2018 (SEPA 2018) to identify the productive forest land, defined as a tree growth rate of > 1 m^3^/ha/year. Our analysis thus did not consider protected forest, but protected forest could be included if it has a growth rate of > 1 m^3^/ha/year. The selection of the 11 study areas was constrained by the limited LiDAR coverage available during the initial phase of Sweden’s national LiDAR campaign, when only approximately 10% of the country had been scanned. The LiDAR coverage in the first year of scanning had the objective to create a stratified design across diverse terrain and along the full latitude of Sweden. From this available data, predominantly forested regions were identified to be included in the study areas encompassing a broad spectrum of landscape characteristics (Paul et al. 2023). Importantly, this high-resolution LiDAR enabled the relatively labor-intensive identification, mapping, and separation of ditches from streams—something impossible with existing topographic maps, which omit roughly 70–80% of all types of waterways (Flyckt et al. 2022).

Buffer widths were chosen to represent the typical narrow buffers of Sweden today (4–7 m wide; Ring et al. 2022) that are not ecologically functional (Kuglerová et al. 2024), but also other science-backed recommendations for buffer widths (Sweeney and Newbold 2014; Lind et al. 2019) that are applied within other jurisdictions such as in Canada and parts of the USA (Table S1). The buffer width scenarios were modeled so that when freshwaters were close to each other, overlapping buffers were only counted once. As a result, wider buffers do not necessarily translate into a linear increase in productive forest area transitioning to CCF (Fig. 2). We stress that our approach is meant to infer consequences at a national level and not to make fine-scale property-level recommendations, where variations in topography, soil type, vegetation cover, among others, would define width needed to sustain the functionality and heterogeneity of a riparian buffer.

To understand how forest property size may affect implementation, we calculated the percentage of productive forests that would transition to CCF on properties categorized as having small (2–25 ha), medium (25–200 ha), or large (> 200 ha) amounts of productive forests (Fig. 2), similar to Bakx et al. (2024). The property boundary data were downloaded from the National Land Survey of Sweden (Lantmäteriet 2019) and we excluded forest fragments smaller than two hectares within a property, then calculated the percentage of productive forest land within the buffers of each type of freshwater (Fig. 2). We chose just one buffer width for this analysis, 30-m, as this buffer is included in the definition of Swedish Agency for Marine and Water Management of the area around waterways (HVMFS 2019: 25)—the most ambitious of all riparian forest definitions in relation to water in national and EU documents. The number of properties per study area ranged from 2 to 258 (Table S3). Mean forest size per property spanned from 12.4 to 1050 ha. Minimum forest size per property was consistently around 2 ha, reflecting the lower threshold for ownership in the dataset, whereas maximum sizes ranged from 129 ha up to more than 2100 ha, indicating uneven ownership structure biased toward small owners. Across our 11 study areas, nearly 78% of the forest properties in our dataset fall into the small size class (2–25 ha), while medium-sized properties (25–200 ha) account for about 20%, and large properties (> 200 ha) for the remaining 2%. This distribution is very similar to other studies where the hectares of forest was divided into property size classes in Sweden; namely, Bakx et al. (2024) found 82% of properties were 2–25 ha, 17% were 25–200 ha, and just < 1% were > 200 ha. Thus, the heterogeneous ownership structure across the 11 study areas is consistent with the Swedish forest landscape, where there are numerous small properties alongside a few very large ones.

We used a linear mixed-effects model with random effects to assess how the forest size on a given property and buffer area of different freshwater types affects the percentage of productive forest potentially transitioned to CCF (fixed effects = forest size class and buffer width), while accounting for variability across study areas (random effect = study area). The linear mixed-effects model was applied using the *lmer* function in R (R Core Team 2024) from the *lme4* package (Bates et al. 2015). Post-hoc pairwise comparisons were conducted using Tukey’s Honest Significant Difference test using the *emmeans* package (Lenth 2025) to determine which groups significantly differed (*p* < 0.05).

## Results

### Legislative feasibility: Regulatory policy instruments as barriers or enablers to CCF in riparian buffers

We identified a total of 20 policies (Table 1)  as relevant: seven at the EU level (EU:1–7 include three directives, one regulation and three recommendatory policies), 12 at national level (SE:1–12), and one at the global/international level (GL:1 is the FSC). Our analysis shows that there are barely any hard barriers to the implementation of CCF in forested riparian buffers and few hard enablers.

Two EU Directives, the “Habitats” (EU:6) and “Birds” (EU:7) Directive, and their implementing legislation (SE:1; SE:4) can act as potential barriers for the implementation of CCF in riparian buffers. Although neither Directive is specific to riparian buffers, nor are buffers explicitly mentioned, if a riparian buffer is home to a protected habitat or species, any active management practices, including CCF, might be prohibited. The EU Water Framework Directive (WFD, EU:1) is not specific to the implementation of CCF, but it can become a hard enabler for the implementation of buffers in general around freshwater because of its binding goals of good status. Since the WFD does not cover streams with catchment areas smaller than 10 km^2^ and lakes smaller than 1.0 km^2^, it excludes many of the streams, lakes and ditches within our study areas. Therefore, the WFD cannot be deemed as being a hard enabler within the Swedish forest context, where over 80% of waterways have less than 10 km^2^ catchment areas and many are not even represented on maps (Flyckt et al. 2022).

The national policy regulating forestry (SE:2, SE:3) and relevant for freshwater in forests does not act as a hard barrier or enabler for CCF implementation in forested riparian buffers. Similarly, the Swedish national policies implementing the WFD in forestry (SE:5; SE:6; SE:7; SE:8; SE:10) do not act as hard barriers nor enablers for the implementation of riparian buffers or CCF management in them, mainly due to their recommendatory character in relation to forested riparian buffer management and protection. While some policies are binding (SE:5), they require additional measures to be decided upon at local–regional level (SE:6), which in their current form (Programs of Measures 2022–2027) do not set any requirements regarding the protection or management of forested riparian buffers. The guidelines for the management of forests in riparian buffers for water quality (SE:12) underline the forest management legacy of buffers and the need for active management for more deciduous trees but do not specify any specific width of the buffer. The policies that do set measurable targets for riparian buffers in forest management (i.e., 15 m in SE:8 and 5–15 m of no-machine zone in SE:11), are guidelines with recommendatory power and their requirements are thus not binding and serve only as soft enablers. FSC certification (GL:1) also acts as a soft enabler. While FSC-certified forest owners do have to abide by specific targets, the voluntary nature of certification entails that it can only be considered a soft enabler.

At both EU and national level, there are guidelines that focus specifically on CCF. At the national level, besides the report on CCF and closer-to-nature forestry (SE:9) that focuses on CCFs applicability, other guidelines implementing the WFD aimed at forestry also emphasize CCF’s potential to attain functional riparian buffers through active management and restoration (SE:10). Similarly, at the EU level, there are guidelines that specifically focus on CCF and closer-to-nature forest management (EU:3). Although the benefits of CCF, as well as the importance of riparian buffers, especially along small streams are underlined, and 30-m buffers are recommended, the guidelines are only recommendations and do not explicitly connect CCF to riparian buffer management. The EU Forest Strategy (EU:2) on the other hand, although not setting measurable targets for riparian buffers, does raise CCF as a suitable management approach for restoration purposes.

The strongest legislative enabler for the implementation of CCF in riparian buffers of productive forests is the recently enacted EU Nature Restoration Regulation (EU:5), hereafter Restoration Regulation, since it sets binding and measurable targets for restoration and protection. The Restoration Regulation is not only binding but also applied in its entirety within all member states, making it coercive and rigid in its implementation (see Treib et al. 2007 regarding the classification of EU policies). It also sets detailed and measurable goals that all member states must achieve. Namely, EU member states should “(…) place effective and area-based restoration measures with the aim to jointly cover, as a Union target, throughout the areas and ecosystems within the scope of this Regulation, at least 20% of land areas (…) by 2030, and all ecosystems in need of restoration by 2050” (Art. 1, 2; EU:5). Also, the Restoration Regulation requires that restoration measures are applied by 2030 to at least 30%, by 2040 at least 60% and by 2050 at least 90% of the total area of all habitat types listed in Annex I that are deemed not in good condition.

The Restoration Regulation can be seen as a hard enabler for the implementation of CCF in riparian buffers around freshwater, since it is binding and establishes riparian buffers as a specific restoration measure (Annex VII, (9) EU:5) and mentions the application of CCF to enhance diversity in ecosystems (Annex VII, 14). Moreover, the regulation lists several aims and measures that can be achieved through CCF, including increasing the “share of forests with uneven-aged structure” (Art. 12, (3) c, EU:5), increase “forest connectivity” (Art. 12, §3 d), increase “tree species diversity” (Art. 12, (3) g EU:5), “[i]mprove connectivity across habitats to enable the development of populations of species, and to allow for sufficient individual or genetic exchange as well as for species’ migration and adaptation to climate change” (Annex VII, (22), EU:5); “[a]llow ecosystems to develop their own natural dynamics for example by abandoning harvesting and promoting naturalness and wilderness” (Annex VII, (23), EU:5). Thus, the Restoration Regulation not only avoids setting barriers for the implementation of CCF in riparian buffers but also holds the potential to act as an enabler for its application.

Our analysis shows that the implementation of CCF is feasible from a legislative perspective since there are hardly any soft or hard barriers to its implementation. Contrarily, both EU and national policies with recommendatory power act as soft enablers for implementation, and the Restoration Regulation is a hard enabler. It is important to note that the legislative feasibility of CCF will depend on how member states, in this case Sweden, decide to implement the EU Nature Restoration Law and on how the management of production forests and riparian buffers is approached in Sweden’s National Restoration Plans.

### Technical feasibility: Ability of CCF in riparian buffers in productive forests to attain policy goals

**Fig. 3:**
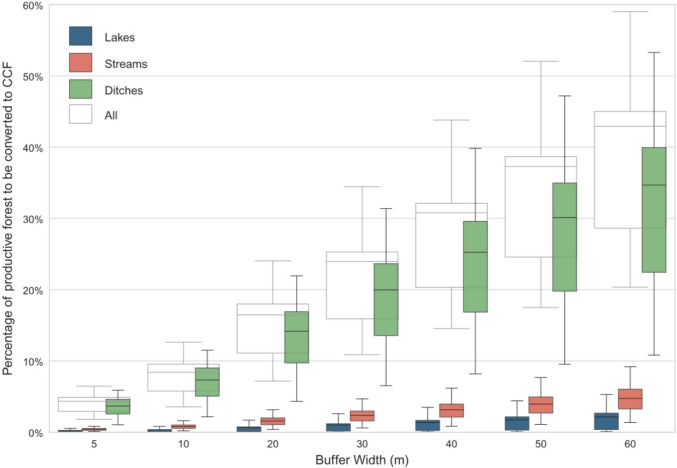
Percentage of productive forest land within the study areas contained within the different buffer widths that could be converted to CCF management. Blue bars show the range and percentage of productive forests included in buffers around lakes; orange bars show the range around streams, green bars show the range around ditches, and white bars show all of these freshwaters combined. The solid line in box plots is the median value, box extents are the interquartile range (IQR), and whiskers show the 1.5IQR value. Analysis available in Table S2, Supplementary material

**Fig. 4:**
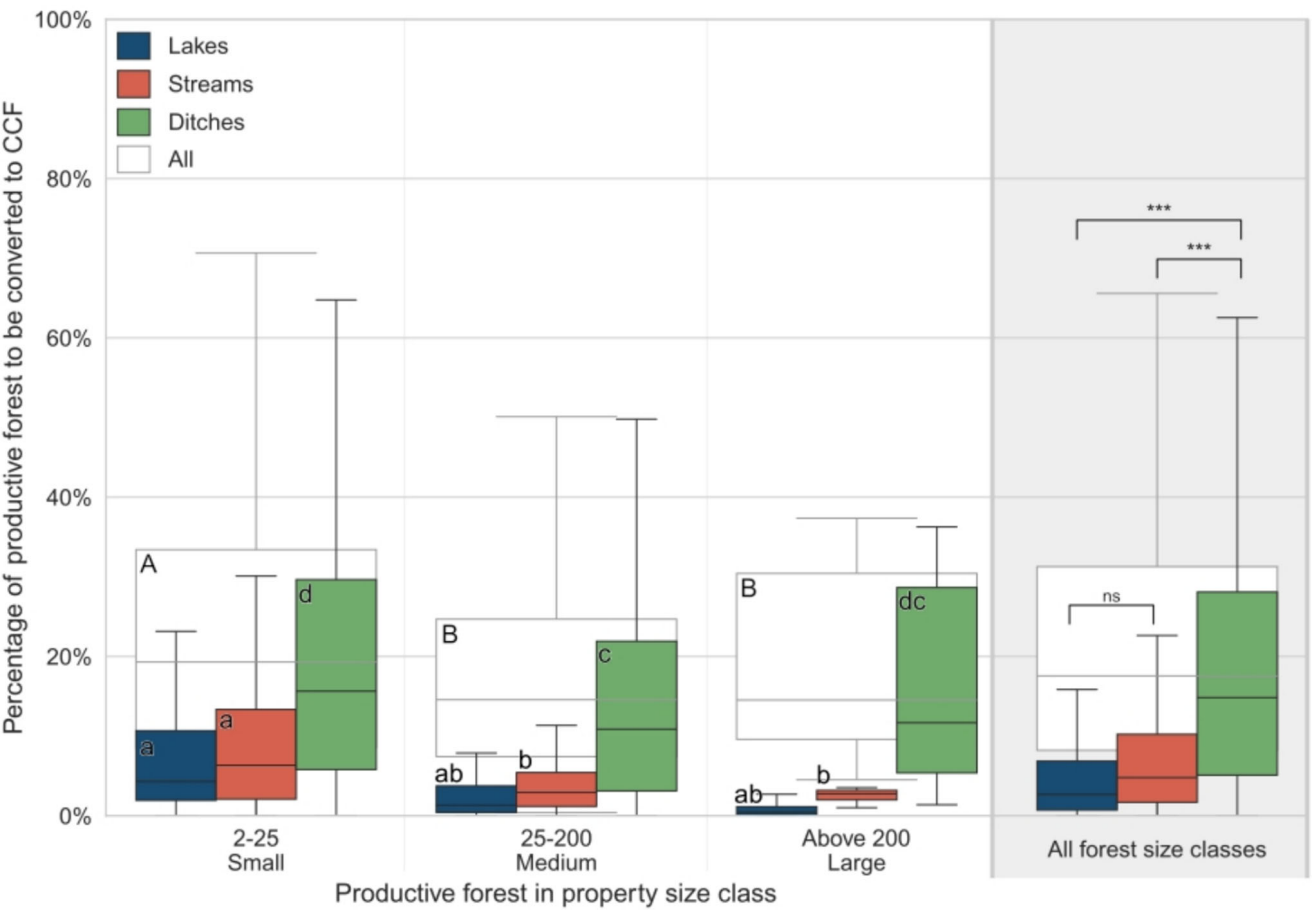
Percentage of productive forest land per property size class estimated to transition to CCF by the hypothetical implementation of 30-m riparian buffers around lakes (blue), streams (orange), forest ditches (green). White boxes represent all data combined. Different upper-case letters denote statistically significant differences between size classes (*p* < 0.05), and different lower-case letters indicate statistically significant difference between types of freshwaters (*p* < 0.05). The gray area on the right shows the aggregated data; lines and *** show statistical differences between 30-m riparian buffers along different types of freshwaters (*p* < 0.05; ns = not significant). The solid line in box plots is the median value; box extents are the interquartile range (IQR) and whiskers show the 1.5IQR value. Analysis available in Table S2, Supplementary material

We found that implementing CCF within riparian buffers around ditches, streams, and lakes combined would represent an estimated transition of 4–42% of the productive forest area in our study areas that are under traditional clear-cutting management (Fig. 3). The variation in transition rates depended on whether a 5-m or 60-m-wide buffer was used, with the 30-m buffer (as recommended by research and policy) having the potential of including up to 23% of productive forests. Specific results differed widely across the three freshwater types studied. Ditches are more numerous than natural streams in the landscape, leading to a higher percentage of productive forests that would be included in their riparian buffers. For example, up to 35% of productive forest could be included around ditches with a 60-m buffer. In contrast, if only natural streams were buffered with CCF-managed forests, less than 5% of productive forests would be transitioned, even in a 60-m buffer scenario (Fig. 3). Different buffer widths around lakes would have a very low effect on the conversion of productive forest to CCF, representing almost no change, reaching just 1% even with a 30-m buffer. These results highlight that the technical feasibility of CCF practices near water will be highly linked to the type of freshwater that would be the subject of future legislation (Fig. 4).

Property size strongly influenced the proportion of productive forests that would be transitioned by a 30-m riparian buffer scenario. Small properties had a significantly higher percentage of productive forests within buffer areas compared to medium and large properties (Table S2; Supplementary material). This is largely due to lakefront parcels often being subdivided into smaller estates. While the percentage of productive forest land around ditches was consistent across all property sizes, owners of small estates would see larger transitions to CCF near natural streams, with nearly 10% of their productive forest potentially converted, compared to less than 5% for medium and large properties. Expected impacts on different ownership classes is therefore a key factor to the feasibility of CCF along riparian buffers in Sweden.

## Discussion

Our analysis of EU, national, and global policies identified that there are few hard and soft barriers to the implementation of CCF in productive forest riparian buffers in Sweden and there are soft enablers present both at EU and Swedish national level. Although reports and guidelines highlight the applicability of CCF and its potential to achieve functional riparian buffers through active management and restoration, especially around small streams, they are only recommendatory and do not always explicitly link CCF to riparian buffer management. Legislation such as the EU WFD and Swedish forest policies do not act as strong enablers, partly due to Sweden’s forestry regulation, and WFD implementation for freshwater in forests (Appelstrand 2007; Keskitalo and Petterson 2012; Johansson and Keskitalo 2014; Hasselquist et al. 2020), and partly due to the size thresholds within the WFD for regulating catchments and lakes.

We found the EU Nature Restoration Regulation to be the only hard enabler for implementing CCF in riparian buffers around lakes, ditches, and streams. This is not surprising since EU regulations set binding (hard) and measurable requirements (Treib et al. 2007). The regulation’s relevance for forested riparian buffers is two-fold. First, the implementation of CCF in riparian buffers and its potential to lead to more natural, uneven-aged habitats with diverse species, could be counted toward the 20% target of the Restoration Regulation for all land areas. When CCF is implemented in riparian buffers on habitats listed in Annex I, these lands can be counted toward the restoration measures required to be put in place for 30%, 60%, and 90% of the areas of those specific habitats by 2030, 2040, and 2050, respectively. Second, the management of riparian forests through CCF could be seen as also contributing to the Restoration Regulation’s goal of restoring all ecosystems that are in need of restoration. In sum, CCF is an eligible instrument to help forest lands in Sweden meet goals for both water quality and landscape restoration.

Our GIS analysis showed that if riparian buffers were managed with CCF between 4 and 42% of Swedish productive forests could transition to CCF, enough to meaningfully contribute to restoration targets. CCF in riparian buffers could allow for a better balance of production and environmental goals, as restoration measures critical to meet biodiversity targets can be implemented while accounting for socioeconomic needs (Chapman et al. 2025). However, if natural streams and lakes are considered as habitats worth buffering and their riparian forest restored, and ditches are excluded, the percentage of productive forests that will be converted into CCF falls to about only 0.5% with a 5-m buffer and just 4.8% with a 60-m buffer, because a significant proportion of the freshwater in productive forests is comprised of ditches or modified streams (Paul et al. 2023). Thus, in the Swedish context, which waters are considered worth protecting is especially relevant, since the lack of buffer on even the smallest waterways can affect downstream waters (Myrstener et al. 2025). In particular, ditches can play an important role in environmental processes (Clifford et al. 2025) changing overall water quality at a catchment level. The aspect of implementing riparian buffers for different types of freshwaters, together with catchment size WFD definitions, brings forth the crucial implications that definitions of habitats and ecosystems may have on the implementation of protection and restoration measures. These definitions also have major implications for the potential of CCF in forest riparian buffers to attain EU policy goals. Definitions are, however, not only ecological but mostly political matters (Head 2019) and ecological processes are not always mirrored in policy requirements.

CCF can also offer a synergistic solution for riparian forest management, especially for smaller properties, and an opportunity for large forest owners. Wider buffers can help mitigate the adverse historic and current effects of forestry on aquatic ecosystems (Olden et al. 2019; Kuglerova et al. 2024; Myrstener et al. 2025) and improve landscape connectivity (Salviano et al. 2021), while alternative forest management within these buffers allows for continued, albeit reduced, timber production. Our analysis of 30-m riparian buffers revealed that while the percentage of productive forest land around ditches was similar across all property sizes, small properties would be the most affected by CCF implementation in riparian buffers, especially around lakes and natural streams. This finding corroborates previous research indicating the disparate implications from CCF implementation depending on the size of their productive forest properties (Bakx et al. 2024). However, our results also show that alternative management in riparian buffers may be a partial solution to the costs and contributions associated with achieving environmental policy goals that are not evenly distributed among landowners or across society at large (Bostedt et al. 2021). Leaving a 30-m buffer zone unmanaged can reduce the total net present value (NVP) of the forest landscape by approximately 4–10%, while CCF with selective logging aimed at promoting broadleaved species within the riparian buffer can reduce these costs to just 1–3% of the total NPV (Sonesson et al. 2021). In specific cases, implementing CCF-managed buffer zones may even lead to an increase in NPV for small holdings (Bakx et al. 2024). The lower economic impact of CCF in riparian buffers—compared to fully set-aside zones—combined with the potential for NPV gains among smaller landowners who are more impacted by the retention of riparian buffers, can also present a compelling economic incentive for adopting wider (> 30m) CCF riparian buffers. On the other hand, large forest owners were less affected by implementing wide riparian buffers and thus, could adopt CCF management with comparatively smaller reductions in harvest. If this pattern holds outside of our study areas, industrial forest owners may be well positioned to play a leading role in implementing more sustainable practices. Although our dataset was weighted toward smaller properties, it still reflects the overall distribution of medium and large sized forest properties in Sweden. Future work could test if using a stratified or ownership-weighted sampling area would influence the outcome. Regardless, our work demonstrates that a single approach may not be universally suitable for all forest property sizes, and that solutions should be tailored to specific contexts.

There are certain caveats when assessing the legislative and technical feasibility of CCF adoption in Sweden that should be addressed by further research. This study did not delve into the expected costs and benefits arising from CCF implementation as well as their distribution among different stakeholders such as landowners, and public and private organizations. Nor did it explore the social and political acceptance of a wide-scale transition to CCF since our study has limited itself to *legislative*, as opposed to political feasibility. While research has shown that social factors considerably restrain a wider implementation of CCF (Hertog et al. 2022), our study aimed to demonstrate the potential of CCF limited to productive forest in riparian buffers to bridge production and protection forest policy goals. Future in-depth exploration of potential economic and political constraints to implementation could shed light on what policy steps are needed if CCF is to be applied more broadly. Also, restoration measures using CCF may help attain some policy goals, but they may also lead to adverse effects in the form of increased emissions or land degradation from driving. Therefore, not only is further research on the effects of CCF needed, but also careful and active interaction between policy sectors to coordinate goals and to avoid an increased gap between policy expectations and actual outcomes (Mårald et al. 2017; Mancheva 2020).

To conclude, CCF-managed riparian buffers in productive forests can reduce impacts on biodiversity and freshwater, maintain timber production, and in that way offer owners of different property sizes varying opportunities for meeting multiple goals with their forest management. CCF in riparian buffers could contribute meaningfully to restoration regulations and water quality goals, but the future feasibility of CCF around freshwater in forests will depend on how EU member states implement the EU Nature Restoration Regulation through their National Restoration Plans, and whether political and economic conditions align with ecological ambition. The choices that Sweden and all other member states make toward implementation may weaken the EU Nature Restoration Regulation’s role as a hard enabler for the implementation of CCF around riparian buffers in productive forests. In the Swedish context, what freshwaters are defined as valuable and in need of protection, would have substantial implications for CCF’s potential to reach EU policy goals. It is therefore essential to account for the varied impacts and trade-offs of alternative management approaches, as well as the multilevel institutional, political, and environmental conditions in each context, since outcomes will differ even under shared regulatory frameworks.

## Supplementary Information

Below is the link to the electronic supplementary material.

Supplementary file1 (PDF 502 KB)

## Data Availability

The authors confirm that all data generated or analyzed during this study are included in this published article and primary and secondary sources and data supporting the findings of this study were all publicly available at the time of submission. The analyzed geospatial data can be accessed through the National Land Cover Database (Naturvårdsverket, Nationella marktäckedata).
