# Supplementary material for: Bridging production and protection: Legislative and technical feasibility of continuous cover forestry around freshwater in Sweden
Source: Ambio. 2026 Jan 23;55(8):1866–81. doi: 10.1007/s13280-025-02340-4 (PMC13319281; doi:10.1007/s13280-025-02340-4)
Supplement: Supplementary file 1 — Supplementary file1 (PDF 502 KB) [file 13280_2025_2340_MOESM1_ESM.pdf]

Supplementary Information: This Supplementary Information has not been peer reviewed.

Title: Bridging Production and Protection: Legislative and Technical Feasibility of Continuous Cover Forestry around Freshwater in Sweden

Table S1. Statistical analysis to assess the percentage of productive forest land within the study area contained within the different buffer widths scenarios.

S.1.1 Results for the type III ANOVA with Satterthwaite approximation (for mixed models).

	Sum Sq	Mean Sq	Num DF	DenDF	F value	Pr(>F)	significance
Buffer width	0.34	0.34	1	200.49	171.22	0.000	***
Waterway type	0.00	0.00	2	201.00	1.04	0.350	ns
Buffer width: waterway type	0.37	0.19	2	200.49	94.17	0.000	***

Significance levels: *** = p < 0.001 | ** = p < 0.01 | * = p < 0.05 | ns = p ≥ 0.1 (not significant)

S.1.2. Pairwise Comparisons of Size Class and freshwater type Using Estimated Marginal Means. Significant different only.

group1. buffer width	group1. waterway	group2. buffer width	group2. waterway	estimate	SE	df	t.ratio	p.value	significance
5	Ditches	20	Ditches	-0.094	0.020	186.0	-4.820	<0.001	***
5	Ditches	30	Ditches	-0.148	0.020	186.0	-7.571	<0.001	***
5	Ditches	40	Ditches	-0.197	0.020	186.0	-10.063	<0.001	***
5	Ditches	50	Ditches	-0.241	0.020	186.0	-12.341	<0.001	***
5	Ditches	60	Ditches	-0.282	0.020	186.0	-14.408	<0.001	***
10	Ditches	30	Ditches	-0.114	0.020	186.0	-5.854	<0.001	***
10	Ditches	40	Ditches	-0.163	0.020	186.0	-8.346	<0.001	***
10	Ditches	50	Ditches	-0.208	0.020	186.0	-10.624	<0.001	***
10	Ditches	60	Ditches	-0.248	0.020	186.0	-12.691	<0.001	***
20	Ditches	40	Ditches	-0.103	0.020	186.0	-5.243	<0.001	***
20	Ditches	50	Ditches	-0.147	0.020	186.0	-7.521	<0.001	***
20	Ditches	60	Ditches	-0.187	0.020	186.0	-9.588	<0.001	***
20	Ditches	5	Lakes	0.117	0.021	186.4	5.661	<0.001	***
20	Ditches	10	Lakes	0.116	0.021	186.4	5.602	<0.001	***
20	Ditches	20	Lakes	0.113	0.021	186.4	5.459	<0.001	***
20	Ditches	30	Lakes	0.110	0.021	186.4	5.301	<0.001	***
20	Ditches	40	Lakes	0.106	0.021	186.4	5.138	<0.001	***
20	Ditches	50	Lakes	0.103	0.021	186.4	4.969	<0.001	***
20	Ditches	60	Lakes	0.099	0.021	186.4	4.796	<0.001	***
20	Ditches	5	Streams	0.125	0.020	186.0	6.381	<0.001	***
20	Ditches	10	Streams	0.121	0.020	186.0	6.182	<0.001	***
20	Ditches	20	Streams	0.113	0.020	186.0	5.781	<0.001	***
20	Ditches	30	Streams	0.105	0.020	186.0	5.376	<0.001	***
20	Ditches	40	Streams	0.097	0.020	186.0	4.962	<0.001	***
20	Ditches	50	Streams	0.089	0.020	186.0	4.540	<0.002	**
20	Ditches	60	Streams	0.080	0.020	186.0	4.112	<0.010	**
30	Ditches	50	Ditches	-0.093	0.020	186.0	-4.770	<0.001	***
30	Ditches	60	Ditches	-0.134	0.020	186.0	-6.838	<0.001	***
30	Ditches	5	Lakes	0.171	0.021	186.4	8.262	<0.001	***
30	Ditches	10	Lakes	0.170	0.021	186.4	8.204	<0.001	***
30	Ditches	20	Lakes	0.167	0.021	186.4	8.060	<0.001	***
30	Ditches	30	Lakes	0.163	0.021	186.4	7.902	<0.001	***
30	Ditches	40	Lakes	0.160	0.021	186.4	7.739	<0.001	***
30	Ditches	50	Lakes	0.157	0.021	186.4	7.570	<0.001	***
30	Ditches	60	Lakes	0.153	0.021	186.4	7.397	<0.001	***
30	Ditches	5	Streams	0.179	0.020	186.0	9.132	<0.001	***
30	Ditches	10	Streams	0.175	0.020	186.0	8.932	<0.001	***
30	Ditches	20	Streams	0.167	0.020	186.0	8.532	<0.001	***
30	Ditches	30	Streams	0.159	0.020	186.0	8.127	<0.001	***
30	Ditches	40	Streams	0.151	0.020	186.0	7.713	<0.001	***
30	Ditches	50	Streams	0.143	0.020	186.0	7.291	<0.001	***
30	Ditches	60	Streams	0.134	0.020	186.0	6.863	<0.001	***
40	Ditches	60	Ditches	-0.085	0.020	186.0	-4.345	<0.004	**
40	Ditches	5	Lakes	0.220	0.021	186.4	10.619	<0.001	***
40	Ditches	10	Lakes	0.218	0.021	186.4	10.560	<0.001	***
40	Ditches	20	Lakes	0.215	0.021	186.4	10.417	<0.001	***
40	Ditches	30	Lakes	0.212	0.021	186.4	10.259	<0.001	***
40	Ditches	40	Lakes	0.209	0.021	186.4	10.096	<0.001	***
40	Ditches	50	Lakes	0.205	0.021	186.4	9.926	<0.001	***
40	Ditches	60	Lakes	0.202	0.021	186.4	9.754	<0.001	***
40	Ditches	5	Streams	0.227	0.020	186.0	11.624	<0.001	***
40	Ditches	10	Streams	0.223	0.020	186.0	11.425	<0.001	***
40	Ditches	20	Streams	0.216	0.020	186.0	11.024	<0.001	***
40	Ditches	30	Streams	0.208	0.020	186.0	10.619	<0.001	***
40	Ditches	40	Streams	0.200	0.020	186.0	10.205	<0.001	***

group1. buffer width	group1. waterway	group2. buffer width	group2. waterway	estimate	SE	df	t.ratio	p.value	significance
40	Ditches	50	Streams	0.191	0.020	186.0	9.783	<0.001	***
40	Ditches	60	Streams	0.183	0.020	186.0	9.355	<0.001	***
50	Ditches	5	Lakes	0.264	0.021	186.4	12.773	<0.001	***
50	Ditches	10	Lakes	0.263	0.021	186.4	12.714	<0.001	***
50	Ditches	20	Lakes	0.260	0.021	186.4	12.571	<0.001	***
50	Ditches	30	Lakes	0.257	0.021	186.4	12.413	<0.001	***
50	Ditches	40	Lakes	0.253	0.021	186.4	12.250	<0.001	***
50	Ditches	50	Lakes	0.250	0.021	186.4	12.081	<0.001	***
50	Ditches	60	Lakes	0.246	0.021	186.4	11.908	<0.001	***
50	Ditches	5	Streams	0.272	0.020	186.0	13.902	<0.001	***
50	Ditches	10	Streams	0.268	0.020	186.0	13.703	<0.001	***
50	Ditches	20	Streams	0.260	0.020	186.0	13.303	<0.001	***
50	Ditches	30	Streams	0.252	0.020	186.0	12.897	<0.001	***
50	Ditches	40	Streams	0.244	0.020	186.0	12.483	<0.001	***
50	Ditches	50	Streams	0.236	0.020	186.0	12.061	<0.001	***
50	Ditches	60	Streams	0.227	0.020	186.0	11.633	<0.001	***
60	Ditches	5	Lakes	0.305	0.021	186.4	14.728	<0.001	***
60	Ditches	10	Lakes	0.303	0.021	186.4	14.669	<0.001	***
60	Ditches	20	Lakes	0.300	0.021	186.4	14.526	<0.001	***
60	Ditches	30	Lakes	0.297	0.021	186.4	14.368	<0.001	***
60	Ditches	40	Lakes	0.294	0.021	186.4	14.205	<0.001	***
60	Ditches	50	Lakes	0.290	0.021	186.4	14.036	<0.001	***
60	Ditches	60	Lakes	0.287	0.021	186.4	13.863	<0.001	***
60	Ditches	5	Streams	0.312	0.020	186.0	15.969	<0.001	***
60	Ditches	10	Streams	0.308	0.020	186.0	15.770	<0.001	***
60	Ditches	20	Streams	0.301	0.020	186.0	15.370	<0.001	***
60	Ditches	30	Streams	0.293	0.020	186.0	14.964	<0.001	***
60	Ditches	40	Streams	0.285	0.020	186.0	14.550	<0.001	***
60	Ditches	50	Streams	0.276	0.020	186.0	14.128	<0.001	***
60	Ditches	60	Streams	0.268	0.020	186.0	13.700	<0.001	***

Significance levels: *** = $p < 0.001$ | ** = $p < 0.01$ | * = $p < 0.05$ | $p \geq 0.1$ (comparisons that are not significant are not shown)

Table S2. Statistical analysis for the percentage of productive forests transitioned to CCF by the hypothetical implementation of 30-meter riparian buffers in forest ditches, streams, and lakes, categorized by percentage of productive forest area on the property.

S.2.1 Results for the type III ANOVA with Satterthwaite approximation (for mixed models).

	Sum Sq	Mean Sq	NumDF	DenDF	F value	Pr(>F)	significance
Property size	1.12	0.56	2.00	1 539.18	25.94	<0.001	***
Waterway	1.23	0.61	2.00	1 536.31	28.31	<0.001	***
Property size:freshwater	0.01	0.00	4.00	1 533.03	0.15	0.960	

Significance levels: *** = $p < 0.001$ | ** = $p < 0.01$ | * = $p < 0.05$ | ns = $p \geq 0.1$ (not significant)

S.2.2. Pairwise Comparisons of Size Class and waterway Type Using Estimated Marginal Means. Significant different only.

group1. property size	group1. waterway	group2. property size	group2. waterway	estimate	SE	df	t.ratio	p.value	significance
Small	Lakes	Medium	Lakes	0.07	0.02	1 535.74	3.20	0.04	*
Small	Streams	Medium	Streams	0.06	0.02	1 536.68	4.03	<0.001	**
Small	Ditches	Small	Lakes	0.12	0.01	1 535.61	7.91	<0.001	***
Small	Ditches	Medium	Lakes	0.19	0.02	1 539.96	9.97	<0.001	***
Small	Ditches	Small	Streams	0.11	0.01	1 539.61	10.28	<0.001	***
Small	Ditches	Medium	Streams	0.17	0.01	1 539.54	11.97	<0.001	***
Medium	Lakes	Small	Streams	-0.08	0.02	1 538.04	-4.08	<0.001	**
Medium	Ditches	Large	Lakes	0.14	0.04	1 534.91	3.25	0.03	*
Large	Ditches	Medium	Lakes	0.13	0.04	1 539.77	3.35	0.02	*

Significance levels: *** = $p < 0.001$ | ** = $p < 0.01$ | * = $p < 0.05$ | $p \geq 0.1$ (comparisons that are not significant are not shown)

Table S.3 Descriptive statistics of the property size classes (based on hectares of productive forest) in the 11 study areas. The study area numbers generally correspond to a north to south gradient, 1 being the most northern and 11 being the most southern study area.

**Study area**	**Number of properties**	**Mean size (ha of forest)**	**Min (ha of forest)**	**Max (ha of forest)**	**Small 2–25 ha (n)**	**Medium 25–200 ha (n)**	**Large >200 ha (n)**
1	75	29.47	2.01	601.69	62	11	2
2	114	21.20	2.32	208.68	92	21	1
3	2	1055.52	3.62	2107.42	1	0	1
4	89	63.85	2.06	1282.25	55	30	4
5	258	28.81	2.01	471.30	191	61	6
6	9	57.48	2.03	458.69	8	0	1
7	180	12.44	2.01	185.51	155	25	0
8	87	25.36	2.04	257.67	64	22	1
9	39	22.49	2.03	176.50	31	8	0
10	58	20.51	2.28	129.36	42	16	0
11	91	27.15	2.01	1048.37	79	10	2
**All sites**	**1002**	**124.03**	**2.01**	**2107.42**	**780**	**204**	**18**

S.4 List of analyzed policies.

Swedish national level:

SE:1 Environmental Code (Miljöbalken, SFS: 1998:808), https://www.riksdagen.se/sv/dokument-och-lagar/dokument/svensk-forfattningssamling/miljobalk-1998808_sfs-1998-808/ (last accessed 10th June 2025)

SE:2 Forestry Act 1979 and 1993 amendment (Skogsvårdslag, SFS; 1979:429; Skogsvårdsförordning, SFS: 1993:1096), https://www.riksdagen.se/sv/dokument-och-lagar/dokument/svensk-forfattningssamling/skogsvardsforordning-1979791_sfs-1979-791/, https://www.riksdagen.se/sv/dokument-och-lagar/dokument/svensk-forfattningssamling/skogsvardsforordning-19931096_sfs-1993-1096/ (last accessed 10th June 2025)

SE:3 The Swedish Forest Agency Regulation and general guidelines concerning the Forestry Act (Skogsstyrelsens föreskrifter och allmänna råd till Skogsvårdslagen, SKSFS 2011:7), https://www.skogsstyrelsen.se/globalassets/lag-och-tillsyn/foreskrifter-efter-amne/skogsvard/sksfs-2011-7-skogsstyrelsens-foreskrifter-och-allmanna-rad-till-skogsvardslagen-.pdf (last accessed 10th June 2025) and ammendments on forest water protection (Föreskrifter om ändring i Skogsstyrelsens föreskrifter och allmänna råd, .SKSFS 2011:7 till Skogsvårdslagen, SKSFS 2013:2), https://www.skogsstyrelsen.se/globalassets/lag-och-tillsyn/foreskrifter-efter-amne/skogsvard/sksfs-2013-2-foreskrifter-om-andring-i-skogsstyrelsens-foreskrifter-och-allmanna-rad-sksfs-2011-7-till-skogsvardslagen.pdf (last accessed 10th June 2025)

SE:4 Species Conservation Regulation (Artskyddsförordning, SFS 2007:845), https://www.riksdagen.se/sv/dokument-och-lagar/dokument/svensk-forfattningssamling/artskyddsforordning-2007845_sfs-2007-845/ (last accessed 10th June 2025)

SE:5 Government decree on the management of the aquatic environment quality (Vattenförvaltningsförordning, 2004:660), https://www.riksdagen.se/sv/dokument-och-lagar/dokument/svensk-forfattningssamling/vattenforvaltningsforordning-2004660_sfs-2004-660/ (last accessed 10th June 2025)

SE:6 Programs of measures (PoM) for the five Swedish Water Districts (Åtgärdsprogram, 2022-2027), https://www.vattenmyndigheterna.se/atgarder/atgardsprogram.html#:~:text=Vart%20sj%C3%A4tte%20%C3%A5r%20tar%20vattenmyndigheterna,och%20kommuner%20ska%20s%C3%A4tta%20in (last accessed 10th June 2025)

SE:7 Guidance for heavily modified water bodies (Vägledning om kraftigt modifierat vatten (KMV) och ekologisk potential, HVMFS 2023:12), https://www.havochvatten.se/data-kartor-och-rapporter/rapporter-och-

andra-publikationer/publikationer/2023-12-07-vagledning-om-kraftigt-modifierat-vatten-kmv-och-ekologisk-potential.html (last accessed 10th June 2025)

SE 8 Guidelines for measure-planning and norm setting for the forest sector's diffuse pollution (Vattenmyndigheternas riktlinjer för skogsbrukets påverkan på konnektivitet och morfologiskt tillstånd: Åtgärder och undantag, Vattenmyndigheterna i samverkan), https://viss.lansstyrelsen.se/ReferenceLibrary/55071/Metod%20HYMO%20p%C3%A5 (last accessed 10th June 2025)

SE: 9 Possibilities for CCF in forestry and definition of closer to nature forestry in Sweden (Förutsättningar för hyggesfritt skogsbruk och definition av naturnära skogsbruk i Sverige, 2023:16), https://skogsstyrelsen.se/globalassets/om-oss/rapporter/rapporter-2023/rapport-2023-16-forutsattningar-for-hyggesfritt-skogsbruk-och-definition-av-naturnara-skogsbruk-i-sverige-.pdf (last accessed 10th June 2025)

SE:10 Agency regulation for classification and environmental quality objectives for surface water (Havs- och vattenmyndighetens författningssamling, HVMFS 2019:25), https://www.havochvatten.se/vagledning-foreskrifter-och-lagar/foreskrifter/register-vattenforvaltning/klassificering-och-miljokvalitetsnormer-avseende-ytvatten-hvmfs-201925.html (last accessed 10th June 2025)

SE:11 Strategic Objectives for Environmental Consideration in Forestry (Nya och reviderade målbilder för god miljöhänsyn: Skogssektorns gemensamma målbilder för god miljöhänsyn vid skogsbruksåtgärder, SKS 2016:12), https://shop.skogsstyrelsen.se/sv/publikationer/rapporter/rapport-2016-12-nya-och-reviderade-malbilder-for-god-miljohansyn.html (last accessed 10th June, 2025)

SE:12 Blue-Yellow-Green objective classification report (Blå-Gul-Grön Målklassning, 2019:18), https://www.havochvatten.se/data-kartor-och-rapporter/rapporter-och-andra-publikationer/publikationer/2019-12-06-bla-gul-gron-malklassning---ett-satt-att-varna-vatten-med-hoga-naturvarden.html#:~:text=Med%20metoden%20Bl%C3%A5%2DGul%2DGr%C3%B6n,%2D%2C%20natur%2D%20och%20fiskev%C3%A5rd (last accessed 10th June 2025)

European Union level:
EU:1 Water Framework Directive (Directive 2000/60/EC), https://eur-lex.europa.eu/eli/dir/2000/60/oj (last accessed 10th June 2025)

EU:2 Forest Strategy 2030 (COM/2021/572), https://commission.europa.eu/document/cf3294e1-8358-4c93-8de4-3e1503b95201_en (last accessed 10th June 2025)

EU:3 Guidelines on Closer-to-Nature Forest Management (Directorate-General for Environment), https://data.europa.eu/doi/10.2779/731018 (last accessed 10th June 2025)

EU:4 Guidelines on Biodiversity-Friendly Afforestation, Reforestation and Tree Planting (Directorate-General for Environment), https://data.europa.eu/doi/10.2779/731 (last accessed 10th June 2025)

EU:5 EU Regulation on Nature Restoration (Regulation 2024/1991), http://data.europa.eu/eli/reg/2024/1991/oj (last accessed 10th June 2025)

EU:6 The Habitats Directive (Directive 92/43/EEC), https://eur-lex.europa.eu/legal-content/EN/TXT/?uri=CELEX%3A01992L0043-20130701 (last accessed 10th June 2025)

EU:7 The Birds Directive (Directive 2009/147/EC), https://eur-lex.europa.eu/legal-content/EN/TXT/?uri=CELEX:32009L0147 (last accessed 10th June 2025)

Global level:
GL: The FSC National Forest Stewardship Standard of Sweden, https://www.se.fsc.org/se-sv/regler/skogsbruksstandard (last accessed 10th June 2025)
